# Improving reporting of health estimates

**DOI:** 10.2471/BLT.16.179259

**Published:** 2016-07-01

**Authors:** Gretchen A Stevens, Daniel R Hogan, Ties Boerma

**Affiliations:** a Department of Information, Evidence and Research, World Health Organization, avenue Appia 20, 1211 Geneva 27, Switzerland.

In May this year, the World Health Organization (WHO) published *World health statistics 2016*.^1^ This report presents the most recent monitoring data on health-related targets of the sustainable development goals (SDGs), and summarizes data availability and data gaps. For more than half of 35 health-related indicators considered in the report, data were available for less than 75% of countries, and for four indicators, data were available for less than 40% of countries. Yet for many of the indicators where there is incomplete data availability, WHO, other United Nations agencies and academic institutions publish global estimates that include country-specific estimates. The production of health estimates has increased tremendously in recent years. The increase is mainly driven by a growing demand to have recent health statistics for key indicators, such as those that were used to measure progress towards the Millennium Development Goals. 

For countries without data or with data of insufficient quality, estimates are calculated using statistical models. Modelling is done to make optimal use of lower-quality or incomparable data, and to fill data gaps. These estimates are primarily used for global monitoring and priority setting by global agencies including donors.^2^ Without information on the input data and methods used to calculate estimates, users may not know whether the estimates are fit for purpose.

To facilitate the appropriate use and interpretation of health estimates, WHO convened a working group to develop the *Guidelines for Accurate and Transparent Health Estimates Reporting* (GATHER).^3^^,^^4^ These guidelines aim to ensure that the documentation of input data and methods used to generate estimates are sufficient for users to evaluate the results and determine whether these may be used for a particular purpose. GATHER provides guidance on reporting quantitative population-level estimates of indicators of health status, health behaviours and health exposures. GATHER is a checklist of 18 items that are considered minimum essential documentation that should be reported every time a new set of health estimates are published. 

The checklist items are designed to communicate data and methods to a broad range of audiences, who may have varying levels of interest in the statistical methods used. Compliance with the checklist should assist users in understanding the input data used and any limitations of the data. For example, GATHER requires that authors provide information on all data sources that were used, such as the population represented, year of data collection and citation for the source. Using this information, a user can evaluate whether a particular estimate is based on a large quantity of high-quality data or a few low-quality studies. GATHER also provides guidance on reporting the limitations of an analysis. It requires that modelling assumptions or data gaps that affect interpretation of the results should be summarized in plain language.

Other reporting items are meant to allow other researchers to scrutinize the data and methods. GATHER requires that authors make all data sets available. This requirement will maximize the benefits of assembled data sets by ensuring that data can be used for other research purposes. GATHER also requires that authors state how source code can be accessed. The requirement to make code available was considered essential to advance the science of health estimation by allowing researchers to build upon one another’s methods and to increase confidence in estimates by enabling external scrutiny.

GATHER’s requirements on reporting input data and source code are ambitious in comparison to other leading guidelines in the medical field. Of the reporting guidelines for main study types found on the EQUATOR website – a clearinghouse for reporting guidelines – eight involve data analysis. Of these, only the *Preferred Reporting Items for Systematic Reviews and Meta-analyses* (PRISMA) requires reporting that would potentially allow reconstruction of the data used.^5^ None of the eight guidelines require that data or code be made available, though some encourage authors to do so.^6^ GATHER’s requirements are consistent with a growing movement among scientists, funding agencies and journals to improve reproducibility of research and reduce waste in research by making data and methods accessible.^7^^–^^9^

Sharing data and basic modelling assumptions will be an important step towards a greater understanding of estimates by a larger audience. This change will need to be supported by regular interaction and dialogue between UN agencies, academic institutions and technical staff of ministries of health to ensure the inclusion of all appropriate input data and greater understanding of methods, as is done through country consultations carried out by WHO for all its estimates.
